# Advances in Retinal Oximetry

**DOI:** 10.1167/tvst.10.2.5

**Published:** 2021-02-08

**Authors:** Anupam K. Garg, Darren Knight, Leonardo Lando, Daniel L. Chao

**Affiliations:** 1 Shiley Eye Institute, Viterbi Family Department of Ophthalmology, University of California San Diego, La Jolla, CA, USA; 2 School of Medicine, University of California San Diego, La Jolla, CA, USA; 3 Janssen Research and Development, Raritan, NJ, USA

**Keywords:** retinal oximetry, retina, imaging, oxygen saturation, oxygenation

## Abstract

Similar to other organs, the retina relies on tightly regulated perfusion and oxygenation. Previous studies have demonstrated that retinal blood flow is affected in a variety of eye and systemic diseases, including diabetic retinopathy, age-related macular degeneration, and glaucoma. Although measurement of peripheral oxygen saturation has become a standard clinical measurement through the development of pulse oximetry, developing a noninvasive technique to measure retinal oxygen saturation has proven challenging, and retinal oximetry technology currently remains inadequate for reliable clinical use. Here, we review current strategies and approaches, as well as several newer technologies in development, and discuss the future of retinal oximetry.

## Introduction

Blood flow to the retina is tightly regulated in order to maintain adequate oxygen supply for metabolic functions. Retinal blood flow has been shown to be affected in a variety of eye and systemic conditions involving ischemic and degenerative mechanisms, such as diabetic retinopathy,^1^^,^^2^ age-related macular degeneration,^3^ vascular occlusions,^4^ and glaucoma.^5^ Studies showing the central role of oxygenation in the pathogenesis of these diseases have led to the development of methods to better evaluate oxygen saturation in normal and diseased fundi.

Different strategies have been employed to estimate oxygen delivery and saturation in a variety of organ systems, ranging from insertion of microelectrodes in the examined tissue to reflectance spectrometry.^6^ Several of these techniques suffer from practical limitations, many of which are detailed below, and consequently retinal oximetry has not been incorporated as a routine clinical measurement. Effectively, only direct gasometrical analysis of arterial blood and transcutaneous pulse oximetry remain in use, whereas organ-directed modalities, such as retinal oximetry, have proven to be more challenging.

Retinal oximetry is defined as a direct, non-invasive imaging technique that allows for the assessment of retinal blood vessel oxygen saturation. By applying optical principles similar to those used in pulse oximetry, current techniques estimate oxygen levels in the major inner retinal vessels (arterioles and veins visible in fundoscopy) with the aim of being utilized as a standardized parameter of perfusion. Currently, although various devices are being evaluated in research studies, as detailed below, additional data demonstrating clinical usefulness are necessary to justify utilization of retinal oximeters in clinical decision-making. Recent technological advances have allowed the development of automatic retinal oximetry, which may enable the use of this technology in ophthalmic clinical practice in the future. Here, we review previous strategies and products and newer technologies in development, and we discuss the future of development.

## Previous and Current Strategies

As previously reviewed by Beach,^7^ the early path to retinal oximetry was based on the tissue measurements that preceded it. Optical assessments of blood oxygen saturation are possible due to the distinct optical properties of oxygenated and deoxygenated hemoglobin, each of which absorbs different amounts of light at most wavelengths. At isosbestic wavelengths (e.g., 570 nm), absorbance of hemoglobin is not sensitive to oxygen saturation. The optical density can be computed by measuring the light returned just outside of a vessel and then calculating the brightness inside of the blood vessel, with the assumption that the decrease in brightness within the blood vessel is due to absorption of light by blood.^8^ The optical density ratio (ODR) is calculated using the ratio of ODs at two wavelengths and has been demonstrated to have an inverse and approximately linear relationship to oxygen saturation in the retina.^9^ Because of this spectrophotometric principle, multi-wavelength optical imaging is a powerful method to estimate the oxygen levels in retinal blood vessels. An important consideration of this method is that oximeters using the multi-wavelength approach must be calibrated to calculate the conversion from ODR to oxygen saturation (see Hardarson et al.^8^). However, several factors can influence the accuracy of this calibration, such as fundus pigmentation and vessel size (see below); as a result, this calibration is not always perfect and can theoretically lead to measurements below 0% or exceeding 100%. Therefore, the calibration of devices, as well as the use of different correctional algorithms by retinal oximetry manufacturers, must be considered when comparing results across different patient populations or retinal oximetry products.

In 1959, Hickam et al.^10^^,^^11^ first reported measurements of oxygen saturation in the human retina. Briefly, they took fundus photographs using a beam-splitting device allowing for simultaneous exposures at two wavelengths, thus allowing for the computation of ODR. In 1988, François Delori^12^ developed a three-wavelength retinal oximetry method, in which retinal oxygen saturation was determined by the optical density of a blood vessel at three different wavelengths (558, 569, and 586 nm). Two oxygen-insensitive wavelengths were used to internally calibrate the system, and the third wavelength was sensitive to oxygen saturation. Delori compared in vivo measurements of oxygen saturation to in vitro measurements and found that this technique was most accurate when the true oxygen saturation was 50% to 100%, but it overestimated oxygen saturation when the true level was <50%. A four-wavelength device similarly found that the error in measured oxygen saturation increased from ±4% at 83% to ±52% at 0% oxygen saturation.^13^ Adaptation of the three-wavelength model to digital cameras led to the implementation of a dual-wavelength approach. Similar to the three-wavelength method described above, an image splitter is attached to a fundus camera, and images of blood vessels are recorded using a pair of wavelengths combinations, one O_2_-sensitive and another O_2_-insensitive. As detailed below, multiple products that calculate retinal oximetry using the dual-wavelength method are currently commercially available and have been used in several clinical investigations.

## Current Products

There are currently two main commercially available systems that are used for retinal oximetry: the Imedos Vesselmap (Imedos Systems GmbH, Jena Germany; founded in 1996) and the Oxymap T1 (Oxymap ehf, Reykjavik, Iceland; founded in 2002). The Oxymap T1 retinal oximeter, which has thus far been used in the largest number of clinical investigations, has been the focus of a number of validation and clinical studies. The device, which uses two digital cameras and one fundus camera, produces fundus photography in which color-coded outlines of segments of the vascular tree, manually selected by the examiner, indicate the measured oxygen saturation of each artery and vein.^14^

**Figure 1.**
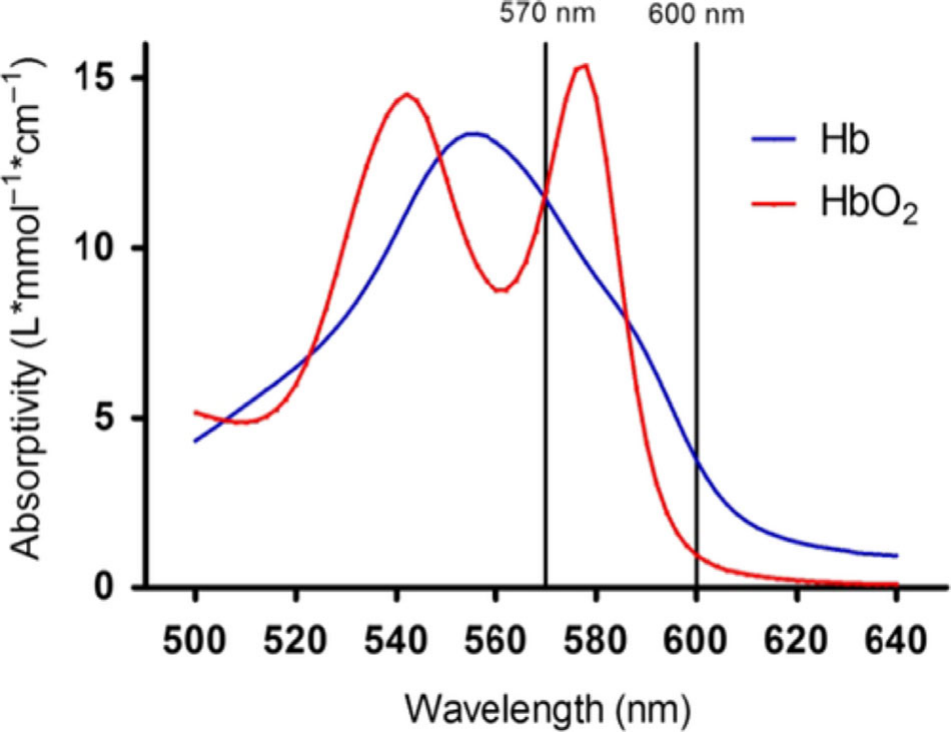
Light absorbance coefficients for oxyhemoglobin (*red line*) and deoxyhemoglobin (*blue line*) at the isosbestic (570 nm) and non-isosbestic (600 nm) operating wavelengths for the Oxymap T1 system. Reproduced with permission from Eliasdottir.^121^ © 2018 Acta Ophthalmologica Scandinavica Foundation. Published by John Wiley & Sons Ltd, based on data from Zijlstra et al.^122^

**Figure 2.**
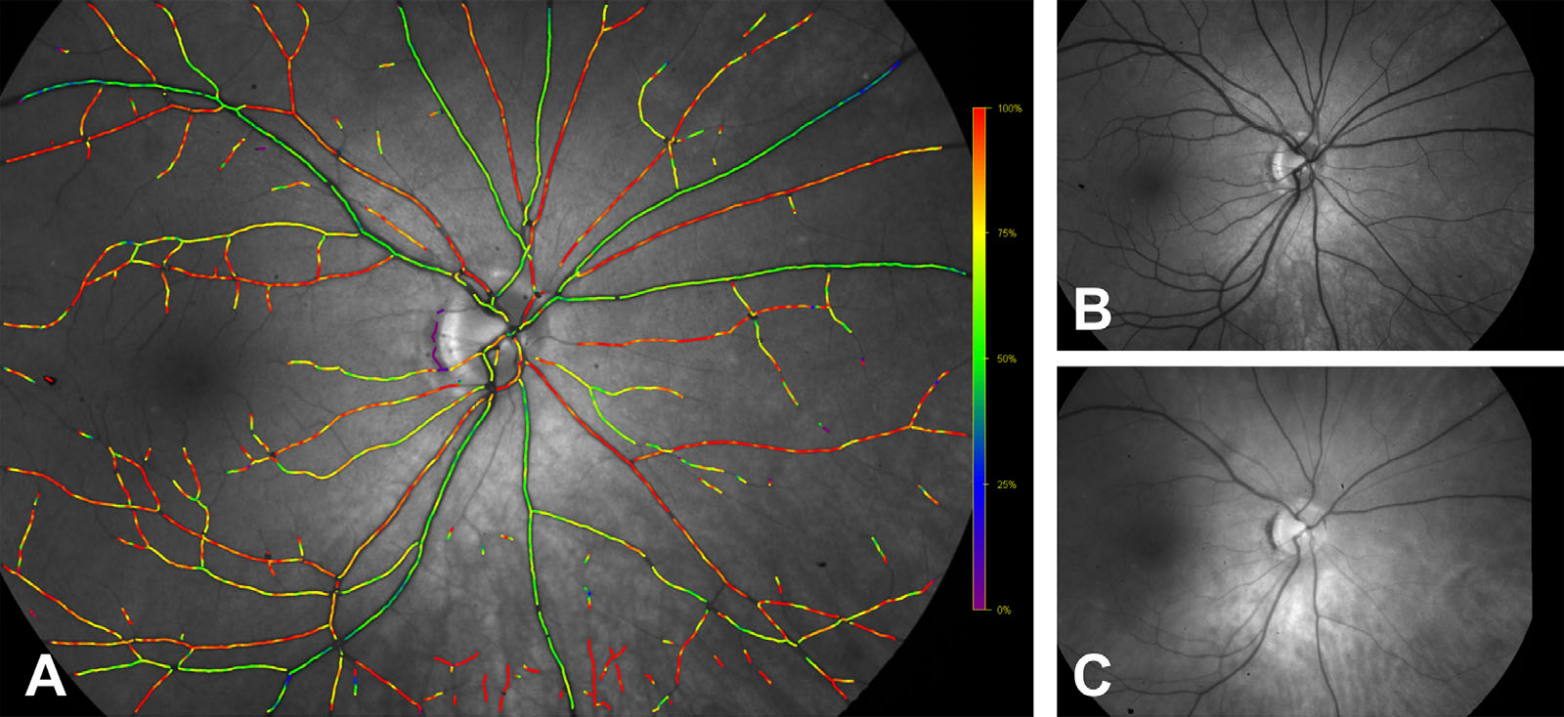
Fundus images registered by Oxymap T1. (A) Color-coded oximetry vessel map generated using split captures at the (B) isosbestic (570 nm) and (C) non-isosbestic (600 nm) wavelengths. Arterioles appear in *red–orange* hues and correspond to O_2_ saturation levels of 90% to 100%; venules are colored in *blue–green*, representing oxygenation in the range of 25% to 50%. Images were provided by Oxymap.

The Oxymap system utilizes the dual-wavelength approach (approximately 570 and 600 nm) (Figs. 1, 2) and has been tested in a variety of clinical conditions. Since its release, the Oxymap has been used by clinical researchers around the world to measure changes in retinal oximetry in a number of diverse ocular diseases such as diabetic retinopathy,^15^ glaucoma,^16^^,^^17^ retinitis pigmentosa,^18^^,^^19^ retinal vein occlusion,^20^ cataracts,^21^ age-related macular degeneration,^22^ ocular trauma,^23^ optic neuritis,^24^ post-surgical status,^25^^,^^26^ high myopia,^27^ Graves ophthalmopathy,^28^ and other systemic conditions such as diabetes without retinopathy,^29^ chronic obstructive pulmonary disease,^30^ myeloproliferative diseases,^31^ Alzheimer's disease,^32^ and multiple sclerosis.^33^

The Oxymap T1 device has shown consistency in normative studies, with the standard deviation of repeated measurements of the same vessel segments measured to be 1.0% in arterioles and 1.4% in venules in a study of 26 healthy patients 18 to 30 years of age.^34^ However, in the absence of ground truth, these results fail to address the accuracy of the measurements, which are limited to vessels and do not address the oxygenation in capillary adjacent areas or anatomical landmarks such as macula and optic disc, where early diseases manifest. Since the introduction of the Oxymap T1, several studies have been conducted in healthy patient populations (several of these studies are listed in Table 1). Notably, comparison of different studies reveals variation in oxygen saturation measurements across different races (Table 1). These differences may be due to methodological differences across studies, as the largest studies to date have each included patients of the same ethnic background.^35^^–^^38^ However, these differences may also be due to differences in the degree of fundus pigmentation, which has been demonstrated to influence retinal oximetry measurements as described above.

**Table 1.** Oxygen Saturation Measurements and Summary of Previous Studies

			Oxygen Saturation (%) ± SD				
Study	Year	PMID	Arterioles	Venules	Wavelengths (nm)	Subjects (s) or Eyes (e)	Comments
Hickam et al.^107^	1963	13961118	N/A	58 ± 10, 60 ± 16	640/800, 640/505	55s, 10s	First non-invasive retinal oximetry assessment in healthy individuals
Schweitzer et al.^108^	1999	10612903	92.2 ± 4.1	57.9 ± 9.9	510/586	30e	Multi-wavelength capture; used method for calibration
Beach et al.^9^	1999	9931217	N/A	55 ± 3.38	569/600	7s	ODR concept
Hammer et al.^43^	2008	19021395	98.0 ± 10.1	65.0 ± 11.7	548/610	20s	—
Kim et al.^109^	2011	21478073	96.0 ± 6.0	54.0 ± 8.0	568/600	11s	Inhalation of 100% O_2_
Mordant et al.^56^	2011	21390065	104.3 ± 16.7	34.8 ± 17.8	420–720	14s	Hyperspectral imaging
Geirsdottir et al.^14^	2012	22786895	92.2 ± 3.7	55.6 ± 6.3	570/600	120s	Cohort of wide age range
Palsson et al.^34^	2012	22395877	93.1 ± 2.3	64.9 ± 3.3	570/600	26s	Cohort of younger patients; saturation varied with change of gaze
Heitmar and Safeen^110^	2012	22395204	P: 94.7 ± 3.9 N: 99.7 ± 3.2	P: 65.1 ± 7.2 N: 90.3 ± 6.7	548/610	12s	Peripheric (P) vs. near-macular (N) arterioles and venules
Man et al.^111^	2013	23802705	94.03 ± 4.89	61.83 ± 4.99	548/610	20s	—
Kristjansdottir et al.^35^	2013	23572109	ODR: 0.22 ± 0.04	ODR: 0.50 ± 0.09	570/600	16s	Choroidal oximetry further assessed
Shahidi et al.^112^	2013	23791637	ST: 103.98 ± 13.07 IT: 96.41 ± 10.89	ST: 48.70 ± 11.47 IT: 47.69 ± 18.06	548/569/586/600/605/610	9s	Hyperspectral imaging; quadrant analysis
Jani et al.^113^	2014	23842102	90.4 ± 4.3	55.3 ± 7.1	570/600	61s	Multiethnic study
Man et al.^114^	2014	24526435	96.02 (IQR, 90.62–98.57)	61.99 (IQR, 56.68–64.08)	548/610	100e	Cohort of different ages
Palkovits et al.^115^	2014	25015353	92.3 ± 3.9	61.8 ± 4.4	545/610	46s	Inhalation of 100% O_2_
Yip et al.^116^	2014	25301879	93.64 ± 6.9	54.22 ± 6.9	570/600	118s	Asian population
Mohan et al.^38^	2015	25923699	90.3 ± 6.6	56.9 ± 6.3	570/600	98s	Asian–Indian population
Yang et al.^117^	2016	26742652	93.2 ± 6.3	60.4 ± 5.3	570/600	126s	Chinese population
Nakano et al.^36^	2016	27434373	97.0 ± 6.9	52.8 ± 8.3	570/600	252s	Japanese population
Liu et al.^37^	2017	27807947	85.5 ± 7.1	48.2 ± 5.5	Not cited	122s	Chinese adolescents

ST, superior temporal; IT, inferior temporal; IQR, interquartile range.

The dual-wavelength strategy of measuring retinal oximetry has also been applied in the Imedos Systems Dynamic Vessel Analyzer (DVA),^39^^,^^40^ which incorporates the Retinal Vein Analyzer (RVA) software and is commercially available for reproducibility research and clinical applications. The DVA module operates on the same spectral principles of oxygenated and nonoxygenated hemoglobin to assess arterial and venous saturation but uses different wavelength cutoff values (610 and 548 nm). The Imedos machine, which is comprised of a fundus camera and a video device, simultaneously captures two fundus pictures and further analyzes retinal oxygen saturation by ratio comparison.^39^ By generating a single full field image, the Imedos oximeter offers the theoretical advantage of avoiding distortions created by methods with image splitters.

The performance of the DVA system has also shown acceptable reproducibility in healthy subjects, with more reliable measurements in retinal venules than arterioles. In a study that included eyes with glaucoma and inherited retinal conditions, more than twofold increased variability in venous oximetry was observed in healthy controls, suggesting more reliable oxygen saturation measurement on the arteriolar level.^40^ One possible explanation for this observation is that this may be due to larger physiological fluctuations in venous oxygen saturations. The authors also pointed out that venules are darker than arterioles, and therefore an equal absolute change in brightness may result in a larger relative change in calculated venous oxygen saturation measurements. Notably, the vast majority of studies listed in Table 1, which include multiple retinal oximetry techniques and devices, have larger standard deviations on their venular measurements, indicating that this finding is not isolated to the DVA system. The DVA and Oxymap systems are compared in Table 2.

**Table 2.** Summary of Commercially Available Two-Wavelength Retinal Oximetry Systems^118^

Name (Manufacturer)	Components	Fundus Camera	Integrated Cameras	Light Wavelengths	Mean Retino-Vascular OS (SD)	Minimum Vessel Diameter	Additional Functions
Oxymap T1 (Oxymap ehf, Reykjavik, Iceland)	Fundus camera, two digital cameras, Oxymap Analyzer software	TRC-50DX (Topcon Corporation, Tokyo, Japan)	Insight IN1800, 1600 × 1200 square pixels (Diagnostic Instruments Inc., Sterling Heights, MI)	600 nm and 570 nm	Arterioles: 98% (3.9%) Venules: 65% (4.1%)	74 µm	—
Dynamic Vessel Analyzer (Imedos GmbH, Jena, Germany)	Fundus camera, digital camera, RVA software	FF450 (Carl Zeiss Meditec, Jena, Germany)	HV-C20A (Hitachi, Ltd., Tokyo, Japan)	610 nm and 548 nm	Arterioles: 92% (2.7%) Venules: 55% (5.6%)	90 µm	Static vessel analyzer, vessel dilatation with flicker

## Limitations of Current Technology

Studies of the dual-wavelength method have shown that several factors, including retinal and choroidal pigmentation, vessel size and thickness, linear blood flow velocity, and retinal nerve fiber thickness, can affect the accuracy of oxygen saturation measurements. Many of these limitations stem from the fact that fundus cameras capture two-dimensional images that introduce several artifacts, such as the influence of pigmentation. These limitations, discussed below, have prevented the widespread adoption of retinal oximetry as it currently stands, necessitated the implementation of correctional algorithms, and ultimately spurred the development of new techniques. Several of the newer technologies in development make use of three-dimensional imaging techniques that reduce the impact of many of these artifacts. However, these pitfalls are important to consider when interpreting the results of current retinal oximetry literature, as they can serve as confounding factors in each of the respective studies.

One limitation of the dual-wavelength oximetry technique is the “central vessel reflex,” in which some vessels can have a hollowed appearance with the central portion of the vessels appearing brighter than the peripheral sections. This effect is pronounced in younger individuals and longer wavelengths. Narasimha-Iyer et al.^41^ described an algorithm that can detect the central vessel reflex that can potentially be used to improved future dual-wavelength oximeters. Previous studies have accounted for this reflex by using a correctional algorithm that averaged the lowest 50% of pixel values inside of blood vessels.^42^ However, the inability to accurately measure oxygen saturation in the central portion of vessels may lead to inaccurate measurements. Furthermore, three-dimensional techniques, as described below, are not affected by this limitation.

A study by Hammer et al.^43^ demonstrated that venous SO_2_ values can range from 74.1% ± 6.6% for individuals with blue irises to 61.7% ± 9.9% for those with brown irises, demonstrating the effect of retinal pigmentation on oxygen saturation measurements. A Monte Carlo simulation of photon transport in the retinal vasculature using six optical wavelengths demonstrated that the reliability of oxygenation values decreased with increasing vessel size and melanin concentration.^44^ Notably, this simulation showed that the relative error of the measured oxygen saturation could be as high as 20% at the highest melanin concentration simulated (8 mmol/L). Similar to the experimental results of Hammer et al.^43^ described above, a Monte Carlo simulation of the dual-wavelength method performed by Rodriguez et al.^45^ also found that vessel saturation was underestimated when melanin concentration was lower and overestimated when melanin concentration was higher.

In the abovementioned study by Rodriguez et al.,^45^ the authors reported the effects of several factors, including incorrect calibration assumptions (of the relationship between ODR and oxygen saturation) and vessel diameter. Notably, in their simulation, the error in calculated oxygen saturation approached 15% as vessel diameter approached 200 µm. The authors demonstrated that this error could be reduced significantly using a correction previously described by Geirsdottir et al.^14^ However, this correction works best for vessel diameters close to the diameter used to calibrate the correction. Consequently, both Oxymap and Imedos (introduced below) have added software to correct for retinal vessel diameter to their products.^46^

Additional factors have been demonstrated to affect retinal oxygen saturation measurements with dual-wavelength imaging. Mohan et al.^47^ studied the correlation of the retinal nerve fiber layer and found a strong positive correlation between perivascular RNFL thickness and concluded that, if artifactual instead of physiological, it may necessitate a correctional algorithm. Patel et al.^48^ simulated light scatter imposed by cataracts using a poly-bead solution and found that light scatter resulted in an artifactual increase in venous ODR. Jeppeson and Bek^49^ demonstrated that measured oxygen saturation was strongly correlated with linear velocity of the blood (as measured by Doppler optical coherence tomography [OCT]). Similar to the above studies on blood vessel diameter, they demonstrated that the application of correctional algorithms can reduce the size of the artifact. Although the authors of these studies concluded that under ideal circumstances the oxygen saturation values calculated by their algorithm were very close to the true value, the study also demonstrated the errors introduced by multiple factors and the need for correctional algorithms.

It is important to note that, although these corrective algorithms improved the accuracy of measurements under ideal circumstances, current retinal oximeters provide only an estimation of retinal oxygen saturation. These techniques can result in errors, as illustrated by measurements that exceed 100% oxygen saturation (see Table 1), which is theoretically impossible. Additional research with newer oximeters and the new technologies described in this review is necessary to demonstrate their reliability at all saturation levels and across a diverse patient population, particularly when they are used in venules and diseases that result in decreased oxygen saturation. These limitations are essential to consider when interpreting the experimental studies described below.

When assessing the potential of a new clinical imaging technique, it is imperative to consider clinical applications. As discussed below, retinal oximetry has been demonstrated to be affected in a variety of clinical conditions; however, these studies rely on averaging several subjects with the disease in question in comparison to an average of control subjects. Although statistically significant differences can be shown in the averages, there are occasional subjects with retinal diseases that have normal retinal oximetry and, similarly, healthy control subjects with decreased retinal oxygen saturation. As they stand, these studies can certainly provide important insight into disease pathophysiology; however, in the absence of a reliable ability to diagnose or track disease progression, the applications of retinal oximetry have remained academic and have not become clinically pertinent.

## Newer Approaches

In order to overcome the limitations of multi-wavelength fundus photography, several new technologies have been in development, including photoacoustic ophthalmoscopy, visible light optical coherence tomography, and hyperspectral imaging. Each of these promising new technologies is described below. Table 3 summarizes the advantages and limitations of each of the technologies described.

**Table 3.** Summary of Alternative Retinal Oximetry Approaches

	Photoacoustic Ophthalmoscopy^64^^,^^71^^,^^119^	Visible Light OCT^74^^,^^78^^,^^79^^,^^83^^,^^120^	Hyperspectral/Multispectral Imaging^51^^,^^53^^,^^55^^,^^56^
Principle	Short laser pulse excitation generates acoustic signals for further image reconstruction.	Low coherence interferometry using broadband illumination (visible spectrum)	Flood illumination causes tissue reflectance, fluorescence, or transmission with different intensities at specific wavelengths.
Advantages	Less influence of pigmentation (melanin) Integrated with OCT, it can provide images with high axial and lateral resolution, retinal vasculature mapping, and flow rate.	Less influence of surrounding tissues in oxygen calculations (accuracy) More continuous detection of blood saturation facilitates calculations Versatility and availability based on current employment of OCT	Nearly real-time information (“in vivo biopsy”) Less influence of vessel thickness May allow small vessel study (capillaries) with multispectral techniques
Limitations	Contact method (patient discomfort and influence on signal noise) Limited adaption to current ultrasound transducer (piezoelectric) May potentially require contrast agents for enhanced image quality (not tested in the eye) Studies so far have focused more on imaging rather than saturation analysis.	Reduced penetration depth and lower sensitivity compared to near-infrared OCT images Patient discomfort and fixation losses due to low contrast of target against light scanning Longer acquisition time caused by dimmer illumination (safety and patient comfort) and increased noise by supercontinuum	Slow image capture (time-sequential imaging systems) Limited depth resolution Oxygen saturation is influenced by light scattering

### Hyperspectral Imaging

Hyperspectral imaging (HSI) uses a hybrid strategy that combines imaging and spectroscopy for the analysis of tissue morphology and function. In HSI, spectral information is added to each pixel of a two-dimensional spatial image to generate a three-dimensional “data cube.”^50^ Medical HSI covers a spectral range from visible to near-infrared light and operates on the reflectance mode in the majority of the machines.^51^

**Figure 3.**
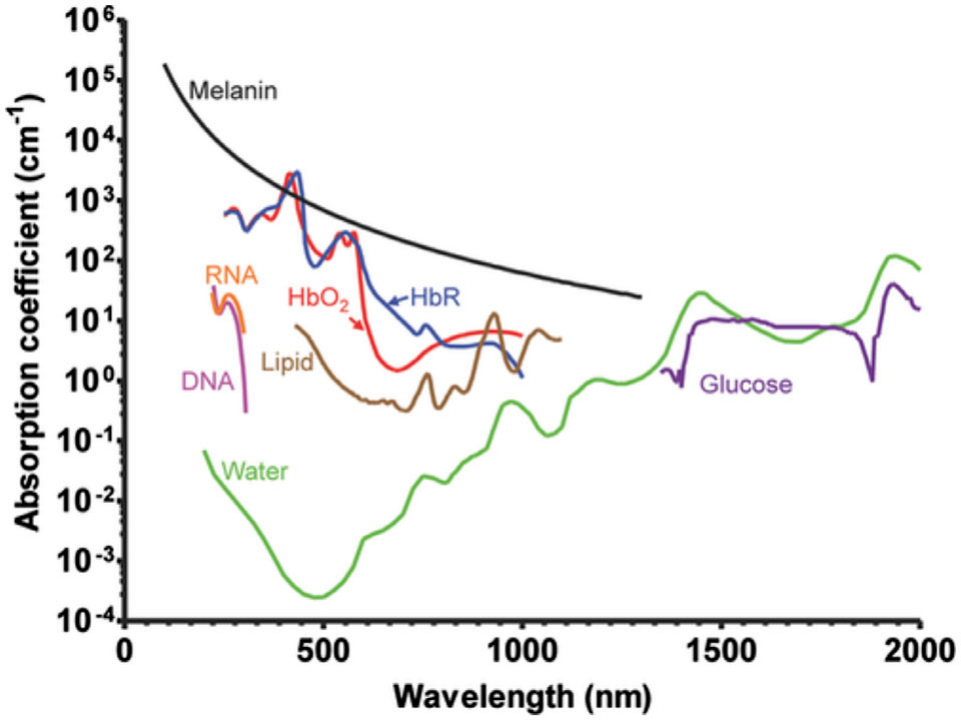
Primary absorbance wavelengths of different endogenous contrast agents demonstrating that the study of hemoglobin using photoacoustic ophthalmoscopy is possible due to its predominant optical absorption compared to other elements, such as water and lipids, in the visible spectral range (380–740 nm). Reproduced with permission from Yao and Wang.^123^ © 2012 by WILEY-VCH Verlag GmbH & Co. KGaA, Weinheim.

Importantly, hyperspectral imaging is fundamentally very similar to the dual-wavelength technique but employs additional wavelengths; however, the fundamental technical limitations are shared between dual-wavelength and hyperspectral imaging. Notably, like dual-wavelength imaging, hyperspectral imaging relies on two-dimensional retinal image and is consequently subject to many of the same artifacts. To measure oxygen saturation of vessels, the spectral components of hemoglobin (oxygenated and deoxygenated) can be analyzed from the vessels captured within the image (see Fig. 3). HSI has been investigated in a variety of organ systems to noninvasively guide diagnosis and interventional treatments. Hyperspectral systems have been used for retinal oximetry since around the 2000s,^52^ with several prototypes being developed since that time. Retinal HSI systems rely on an integrated fundus camera connected to a light source and a detector. One of the main advantages of hyperspectral method compared to two-wavelength imaging is decreased interference by blood vessel thickness and circulating blood cells in oxygen measurements,^53^ although the relatively long time for image registration remains a limiting factor.

HSI has been shown to be applicable to the evaluation of both normal and diseased retinal perfusion, in which a quali-quantitative saturation map is constructed. Patel et al.^54^ tested one HSI system composed by a fundus camera and tunable laser source in six human eyes showing repeatability and reproducibility comparable to other commercially available two-wavelength devices. An improved version of this prototype, based on a hyperspectral camera that can provide multispectral fundus images, was tested with the aim of analyzing oximetric and total hemoglobin in capillary areas outside of large vessels.^55^ Mordant et al.^56^ utilized hyperspectral imaging in 14 normal volunteers and one illustrative patient with a retinal branch artery occlusion to calculate the oxygen saturation of the retinal vasculature. The authors reported that the technique measured lower than normal oxygen saturations in the patient with branch retinal artery occlusion.

Hyperspectral technology has been investigated in vascular and degenerative eye diseases, including diabetic retinopathy, age-related macular degeneration,^57^ glaucoma,^58^ and radiation retinopathy,^59^ among others, with limited non-comparable results. In a 2015 study by Tayyari et al.,^60^ patients with mild and moderate diabetic retinopathy were found to have diminished retinal blood flow and lower arteriovenous difference compared to age-matched healthy controls. Interestingly, the variability of oxygen saturation in the control group was much higher in venules than arterioles, similar to the findings of dual-wavelength systems described above, suggesting that this finding may be due to physiological fluctuations rather than specific to a specific type of oximeter. Two separate studies by Mordant et al.^58^ and Shahidi et al.^61^ have demonstrated higher oxygen saturation in venules of patients with advanced glaucoma compared to healthy or mild-moderate diseased controls.

The ability to compute oximetry maps with high spatial resolution in human patients demonstrates that hyperspectral imaging has the potential to emerge as a clinical retinal oximetry technique. However, as no study has directly compared the results obtained with standard two- or three-wavelength imaging techniques with hyperspectral datasets, additional investigations are necessary to clearly demonstrate the advantages. Limitations of HSI models involve lengthy acquisition and analysis times. Lengthy acquisition times are particularly challenging, as resultant eye movements may affect illumination conditions during imaging.^54^ Furthermore, additional validation is necessary to demonstrate the ability of HSI to provide reproducible results across a variety of retinal properties, such as vessel diameter, pigmentation, and lens opacities.

### Photoacoustic Ophthalmoscopy

**Figure 4.**
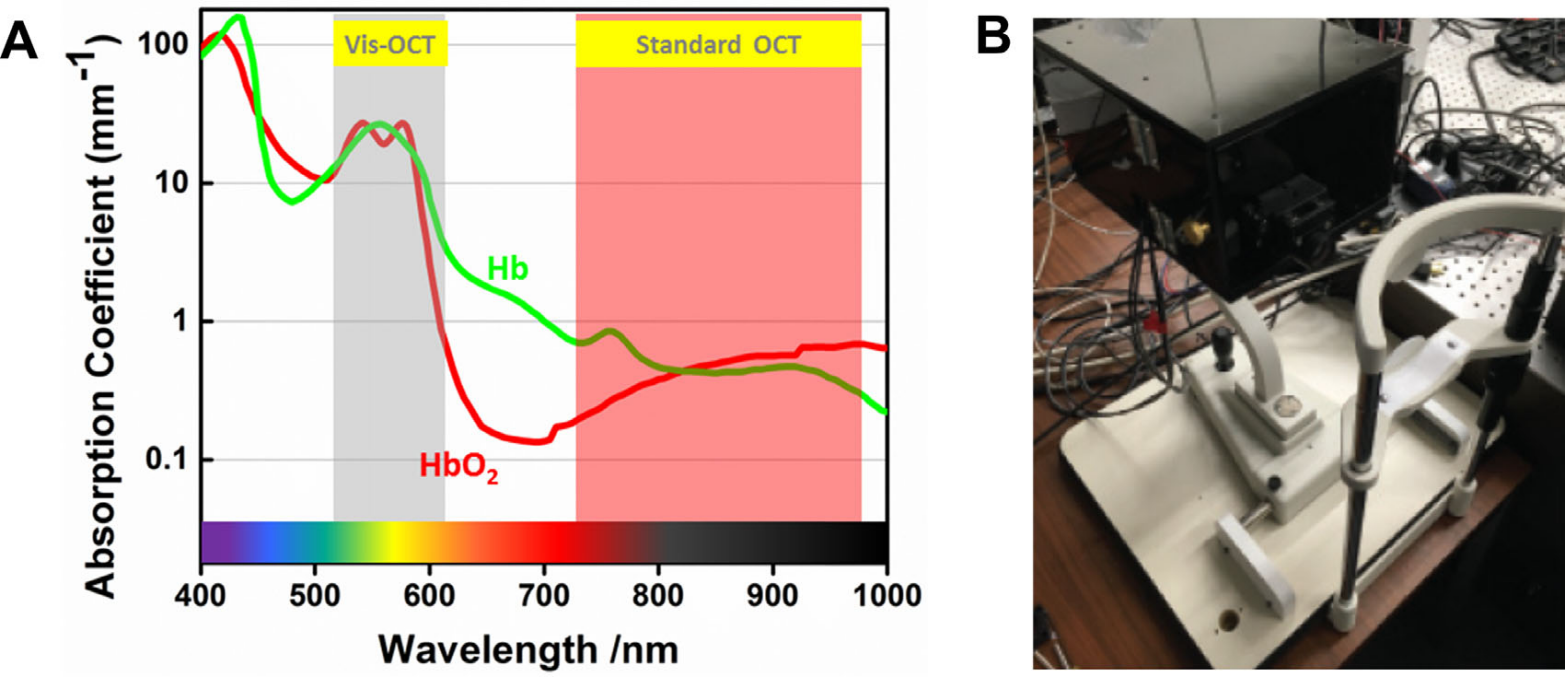
(A) Coefficients of oxygenated (HbO_2_) and deoxygenated (Hb) hemoglobin in logarithmic scale demonstrate the higher extinction coefficients in the visible range responsible for enhanced image quality for the assessment of retinal oximetry. Reproduced from Pi et al.^124^ © 2018 The Optical Society under the terms of the OSA Open Access Publishing Agreement (B) Prototype of visual light optical coherence platform. Reproduced from Chong et al.^75^ © 2016 The Optical Society.

Photoacoustic (PA) imaging has recently emerged as a promising technology for measuring oxygen saturation, offering notable advantages over traditional dual-wavelength imaging (Fig. 4). Short laser pulses are delivered toward a tissue, being both scattered and absorbed within it. Upon meeting an optical absorber in the photon path, the absorbed energy is converted to heat, and wideband and ultrasonic waves are produced as a result of thermoelastic expansion, also known as the photoacoustic effect. The resulting ultrasonic photoacoustic waves are recorded at each location where laser pulses were delivered using an ultrasound transducer in order to generate an image of the studied tissue. The arrival time of the photoacoustic waves is utilized in order to infer the spatial location of the absorbers in the tissue. The magnitude of the resulting photoacoustic waves is proportional to the energy deposition, which is related to the optical absorption properties of various pigments. As a three-dimensional imaging technique, in comparison to dual-wavelength/hyperspectral imaging, which are two-dimensional techniques, PA imaging is not subject to many of the same artifacts.

Because oxygenated and de-oxygenated hemoglobin are two of the most prominent optical absorbers in biological tissues, PA imaging has been applied to the measurement of oxygen saturation. The technology was first demonstrated in vitro by a team of researchers at University College London in 2005.^62^ Subsequently, a study led by Lihong Wang at Texas A&M University demonstrated that PA imaging can be used in human subjects to measure the oxygen saturation in single subcutaneous blood vessels in hypoxia, normoxia, and hyperoxia.^63^ PA imaging has since been demonstrated to be an effective technique to measure oxygen saturation in vivo in a variety of organs, including the brain, ears, esophagus, and colon.^63^^,^^64^

PA anatomic imaging of the eye was proposed by de la Zerda et al.^65^ in 2010. In the following years, several methods aiming to enhance retinal oxygen saturation evaluation focused on integrating techniques to overcome imaging limitations related to acquisition time and scanning area. These strategies included photoacoustic microscopy, multimodal optical scanning, and integrated OCT,^66^^–^^68^ applied to small animal eyes. In integrated OCT and PA imaging, blood flow measured using OCT allows for deduction of retinal oxygen metabolism after deriving the oxygen saturation and vessel diameter using PA imaging.^68^

Multiple studies have demonstrated the ability to use PA ophthalmoscopy for in vivo retinal imaging.^65^^,^^69^ For example, a study by Jiao et al.^69^ in 2010 demonstrated the ability for a stationary transducer in contact with the eyelids (via ultrasound gel) to provide high-resolution (approximately 20 µm in the axial and lateral directions) volumetric imaging of the retinal microvasculature. In 2014, Song and colleagues^68^ demonstrated a technique using PA microscopy to measure retinal oxygen saturation in rats. Briefly, they scanned a circular region surrounding the optic disk at three wavelengths and used the multi-wavelength PA amplitudes to estimate the oxygen saturation at each vessel in this region. Their measurements in normal wild-type rats showed an average arterial and venous oxygen saturation of 93.0% ± 3.5% and 77.3% ± 9.1%, respectively. As described below, they also combined this technique with OCT to compute the metabolic rate of oxygen consumption. Hennen et al.^70^ utilized PA imaging to measure oxygen saturation in the anterior eyes of rabbits, discovering that measurements with this technique were typically lower than those measured with pulse oximetry. The authors achieved an imaging resolution of 70 µm in the lateral direction and 54 µm in the axial direction.

In 2018, Hariri and co-workers^71^ published the first experiment in which the oxygen gradient of the retina and choroid was assessed based on PA ocular imaging. The researchers applied a controlled model of ischemia–reperfusion by continuous intraocular saline infusion and compared fluctuations in fundus oxygen saturation with a peripheral pulse oximeter positioned on the animal's paw. Hariri et al.^71^ utilized PA imaging to measure chorioretinal oxygen gradients, also in rabbits, and found a strong correlation (*R*^2^ = 0.98) between pulse oximetry and PA imaging at different percentages of inhaled oxygen. When intraocular pressure was increased via intraocular phosphate buffered saline injections, the authors reported a sixfold decrease in chorioretinal oxygen saturation with no concurrent changes in the pulse oximetry.

The abovementioned studies demonstrate that PA imaging is a promising technique for the measurement of retinal oxygen saturation, which may overcome some of the potential limitations of dual-wavelength oximetry. The Monte Carlo simulation conducted by Liu et al.^44^ described above that demonstrated the limitations of multi-wavelength retinal oximetry concluded that PA imaging is accurate regardless of vessel diameter or melanin concentrations and thus might be an important tool in the clinical setting. However, as noted by Hariri et al.,^71^ they were unable to measure photoacoustic signals from the pigmented rabbit eye due to the strong photoacoustic signal generated by melanin. However, Hennen et al.^70^ noted that using longer wavelengths in melanin-rich samples may overcome this issue, as melanin has smaller absorption at longer wavelengths. Another important consideration of PA imaging, as described by Wang and Hu,^72^ is that the achievable spatial resolution is proportional to the imaging depth. Consequently, although high-resolution imaging of individual vessels was possible in studies of small rodents, studies of large animals have thus far been unable to assess retinal oxygen saturation with the same precision, which greatly limits its clinical applicability in human subjects. Furthermore, additional limitations, such as the requirement of eye contact for signal detection, use of contrast agents,^64^ need for better refined laser sources, and algorithm-based noise corrections require additional investigation.

### Visible Light OCT

Developed in 1991 by a group of researchers led by James Fujimoto at MIT,^73^ OCT has been widely employed in research and different medical fields and has become a standard imaging tool in ophthalmology. This technology uses near-infrared light to provide cross-sectional and three-dimensional views of the retina and optic disc in vivo, allowing noninvasive structural assessment with high resolution. The ability to generate three-dimensional images, in contrast to the two-dimensional images created by fundus photography, is a key advantage that eliminates many imaging artifacts.

In 2002, following the progresses in the illumination systems, Považay and co-workers were able to successfully develop a different OCT machine that, instead of near-infrared, was based on a newly discovered light source named supercontinuum.^74^ Near-infrared light has been the preferred choice in OCT devices for its deep tissue penetration and facilitated access to manufacturing. The supercontinuum, however, generates OCT-based retinal images through the visible light spectrum with higher-depth resolution and allows for additional applications, including oximetry.

**Figure 5.**
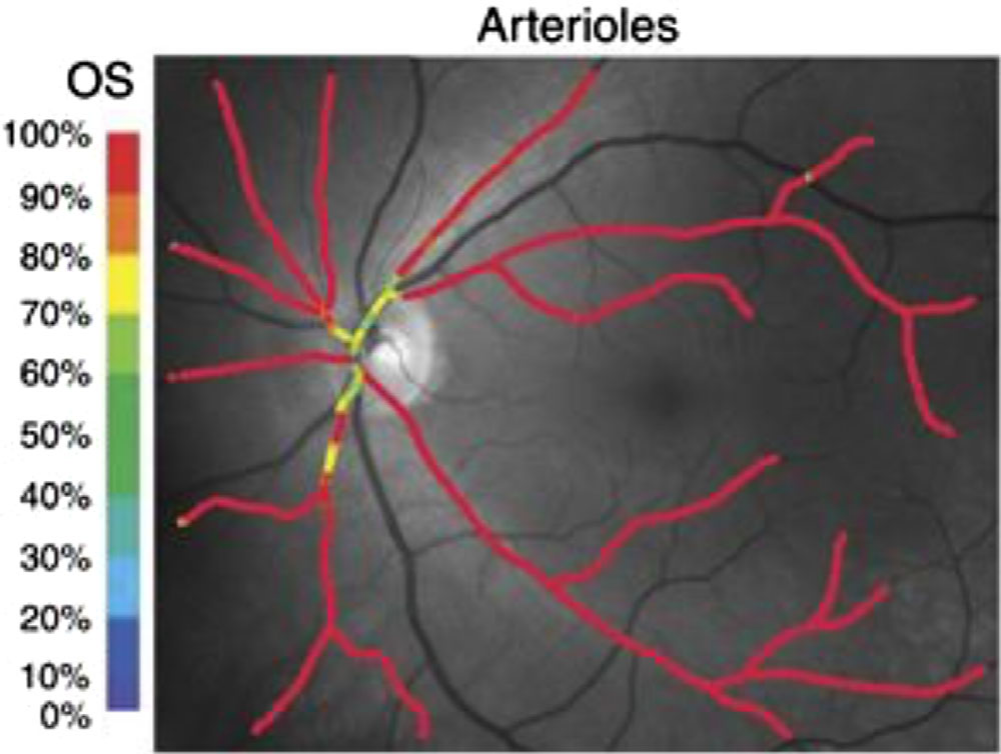
Color-coded oximetry maps using hyperspectral imaging technology display different patterns for comparison in a healthy subject (upper row) and a subject with BRAO (lower row). In this subject with BRAO, lower oxygen saturation is observed in the inferotemporal territory of the retina. Reproduced with permission from Mordant et al.^56^ © 2011 Macmillan Publishers Limited.

Visible-light OCT (vis-OCT) technology, through spectroscopic calculations, allows for the measurement of the oxygen in the blood vessels based on the operating wavelength range (Fig. 5). Although many commercially available OCT systems, such as spectral-domain OCT devices, work on wavelengths around 800 nm to provide good registry of retinal architecture, OCT with visible light can also detect retinal vessels and quantify endogenous chromophores, such as hemoglobin,^75^ in order to calculate estimates of oxygen saturation.^76^ Investigations with vis-OCT for oxygen quantification have been previously conducted in animals under various experimental scenarios.^76^^,^^77^ A 2015 study by Yi et al.^78^ utilized an integrated vis-OCT system guided by scanning laser ophthalmoscopy in four healthy volunteers. The authors demonstrated imaging around the macular region and optic nerve head, revealing a 20% or greater difference in retinal arteries and veins in close proximity to the optic nerve head. A follow-up study from the same group with an additional four patients demonstrated oxygen saturation in major central retinal vessels and utilized a sampling strategy to increase the accuracy of oxygen saturation estimation.^79^ These studies provided a foundation for using vis-OCT for the measurement of retinal oximetry in a clinical setting.

More recently, two studies have been published in 2020 demonstrating the ability to use vis-OCT angiography to measure microvascular oximetry down to the level of retinal capillaries. Pi et al.^80^ utilized vis-OCT angiography to assess oxygen saturation in the retinal capillary beds of rats in response to changes in the inspired concentration of oxygen. Song et al.^81^ performed microvascular oximetry of the capillary network in the foveal region of human volunteer subjects, finding that oxygen saturation was between the retinal arterioles and venules as expected. As vis-OCT expands into other fields such as OCT angiography,^82^ several practical challenges are still being addressed before systems are implemented into clinical practice. These considerations involve portable machines suitable for the hospital setting, adjusted corrections of low signal-to-noise ratios seen in humans compared to animal eyes, laser safety, and light interferences in the measured retinal metabolism.^83^

## Clinical Applications

Noninvasive retinal oximetry is an emerging technology that has been used in research protocols to reliably measure oxygen saturation level in retinal vessels.^7^^,^^8^ As vascular changes have an established or emerging role in the pathogenesis of a number of common retinal pathologies, such as diabetic retinopathy or vessel occlusions, these devices present a number of opportunities for clinical use. With increased understanding of these technologies, these devices and the detailed information provided about retinal oxygenation may improve our understanding of disease pathogenesis and one day improve clinical outcomes.

### Age-Related Macular Degeneration

Age-related macular degeneration (AMD) is a common cause of blindness in the United States and a disease with large societal costs.^84^ The pathophysiology of this disease remains to be fully elucidated; however, some studies suggest that hypoxia and ischemia play a role. Findings have included a positive correlation between retinal venular oxygen saturation and age in patients with exudative AMD,^22^ whereas venular oxygen saturation decreased with age in healthy controls. A newer study by Jakobsen et al.^85^ using the Oxymap retinal oximetry system demonstrated that increasing venular oxygen saturation following a loading dose with anti-vascular endothelial growth factor (VEGF) treatment was independently associated with a 17% higher risk of a unfavorable structural response (measured by change in macular thickness). Notably, oxygen saturation measurements prior to treatment were unable to predict treatment response. Nonetheless, increased venular oxygen saturation in patients with AMD in these studies suggests that retinal oxygen delivery may play a role in disease pathogenesis. However, at this time, the limited amount of data, in addition to the inability to predict treatment response or reliably guide medical therapy, has prevented retinal oximetry from becoming a standard clinical measurement for patients with AMD.

### Diabetic Retinopathy

Diabetic retinopathy is known to be related to biochemical pathway changes caused by prolonged hyperglycemia with subsequent hypoxia-induced changes.^86^^,^^87^ In evaluating the role of retinal oximetry in diabetic retinopathy, it is important to remember the technology's limitations. For example, the devices and studies are unable to calculate the oxygen tension within certain portions of the retina. Additionally, as described above, many factors, such as retinal pigmentation, can influence the retinal oximetry measurements. Using oxygen saturation measurements cannot provide information directly on the mechanism of diabetic vascular changes. However, the findings thus far are supportive of theories that point to blood shunting and bypassing of nonperfused areas resulting in increased oxygenation of venules in the retina. Multiple studies, using both the Vesselmap and Oxymap systems, have demonstrated that retinal oximetry may have predictive value as a supplemental tool in determining the severity of diabetic retinopathy.^88^^,^^89^

Using a commercially available scanning laser ophthalmoscope (Optos 200TX; Optos, Dunfermline, Scotland) combined with commercial software from Oxymap, a recent study by Blair and colleagues^42^ measured retinal oximetry in 46 healthy patients, as well as 135 diabetic patients with and without diabetic retinopathy. Their primary finding was that the retinal venous saturation was increased in non-proliferative diabetic retinopathy, whereas in proliferative diabetic retinopathy the retinal arterial diameter was decreased, and venous oxygen saturation was increased. Another group, led by Kashani et al.,^90^ applied a retinal oximetry device using hyperspectral computed tomographic imaging spectroscopy. This device was added to a conventional fundus camera and was used to measure retinal vascular oxygen content in human subjects. Similar to other studies, arteriovenous difference was significantly lower in patients with proliferative diabetic retinopathy than healthy control subjects (14% vs. 26%). Although these studies demonstrate that venous oxygen saturation is likely increased in diabetic retinopathy, additional data demonstrating a role for retinal oximetry to guide clinical decision-making is likely necessary for retinal oximetry to become practically useful for clinicians.

Other studies have sought to explore the clinical predictive value of retinal oximetry. In a study of 722 patients, Bek et al.^89^ found that venous oxygen saturation contributed more than hemoglobin A1c to retinopathy grade, using either diabetic macular edema or proliferative diabetic retinopathy as an endpoint. However, it is important to note that the authors measured the contributions of several variables, all of which contributed less than 15% of the variation in retinopathy grade. Furthermore, the authors do not address whether they controlled for factors that can influence oxygen saturation measurements, such as fundus pigmentation, when selecting their patient population. Another study by Bek et al.^91^ demonstrated that oxygen saturation in retinal arterioles prior to treatment with an anti-VEGF medication contributed to predicting visual acuity and central retinal thickness after treatment. Their findings validated the finding of increased oxygen saturation in large retinal venules that has been described in prior studies.^92^ Interestingly, although the study by Bek et al.^91^ noted that venous oxygen saturation was increased in patients with diabetic retinopathy (as seen in other studies), the authors did not see a significant decrease in venous oxygen saturation following anti-VEGF treatment, although this study had a relatively small sample size (34 patients with retinal oximetry after treatment). A similar study examined these effects after pan-retinal photocoagulation and concluded that the effects of retinal photocoagulation in diabetic retinopathy was not correlated with changes in oxygen saturation in larger retinal vessels.^93^ It is important to note that these studies did not utilize fluorescein angiography (FA), which is the current gold standard for evaluation of the vascular in diabetic retinopathy. Comparing FA findings with retinal oximetry (as studies have done for retinal vein occlusion, described below) can help to validate retinal oximetry as a clinical measurement. Further, it will be necessary to demonstrate that retinal oxygen saturation measurements can influence clinical decision-making in a meaningful way relative to FA in order for it to become a standard clinical measurement. Combined, these studies suggest that, although increased venous oxygen saturation is seen in patients with diabetic retinopathy, significant changes in oxygen saturation are not necessary to see positive effects of treatment. However, if replicated, the ability to predict treatment response reported by Bek et al. provides a potential clinical application, as retinal oximetry could conceivably be used by clinicians to help provide patients with assistance in selecting treatment options, especially if this predictive value can be demonstrated in additional treatment options and pathologies.

### Branch Retinal Vein Occlusions

Retinal vein occlusions (RVOs) are the second most common cause of vision loss due to retinal vascular disease.^94^ They can be divided into central or branch RVOs depending on the location of the occlusion. As described below, retinal oximetry has demonstrated some potential to help in differentiating between ischemic branch RVO (BRVO) and non-ischemic branch RVO. Yang et al.^95^ utilized the Oxymap retinal oximeter to specifically compare vessels immediately affected by a retinal vein occlusion, normal vessels in the same eye, normal vessels in the opposite eye, and healthy patients. Mean arterial and venous oxygen saturation was significantly higher in healthy individuals. BRVO was further classified into ischemic or non-ischemic using FA. They observed that, in patients with ischemic BRVO, the venous oxygen saturation in the affected vessels was decreased and the arterial saturation was increased. In non-ischemic BRVO, the mean venous oxygen saturation was lower in the affected vein. Notably, this study had a limited number of patients with BRVO (22 total patients; 13 ischemic BRVO and nine non-ischemic patients), with all patients having the same ethnic background. Another study of 24 patients by Hardarson and Stefánsson^96^ found that venular saturation in BRVO was extremely variable, with a range of 12% to 93% oxygen saturation in occluded venules. This study suggested that many factors in BRVO may contribute to the oxygen saturation, including severity of disease and location of the occluded vessel (possibly due to collateral flow). A larger study looked at a cohort of 43 RVO patients to identify a relationship between oxygen saturation and arteriovenous differences in relation to the retinal ischemic index determined using ultra-widefield FA.^97^ This study determined that venous oxygen saturation and arteriovenous difference correlated with ischemic index in patients with central RVO, but no correlation was found for BRVO. These results suggest a possible clinical role for retinal oximetry, as the authors suggest that retinal oximetry may serve as a potential alternative to FA for patients with central RVO. A 2019 study by Nicholson et al.^98^ found that retinal nonperfusion (measured using ultra-widefield angiography) had a negative correlation with arteriovenous difference measured with the Oxymap retinal oximeter. Interestingly, this correlation was significant in the posterior pole but not the peripheral retina. The data suggest that oximetry measurements may potentially provide insight into the severity of an occlusion, though the usefulness of oximetry may be location dependent. Future studies will require larger sample sizes to better establish normative data across patients with varying severity of disease and different levels of fundus pigmentation and allow for more definitive conclusions about the ischemic environment created by RVO.

### Glaucoma

The exact pathogenesis of many glaucoma-related optic neuropathies remains to be completely elucidated. Multiple theories of the pathogenic mechanism exist, but some studies have shown that irregularities in ocular blood flow may have a relationship with neuronal loss.^99^^,^^100^ Other studies have demonstrated that a reduced diastolic perfusion pressure is an important risk factor in primary open-angle glaucoma.^101^ Given the possibility of a vascular etiology, retinal oximetry may serve as a useful tool for gaining a better understanding of the underlying vascular mechanisms in different stages of glaucoma.

In a 2014 study by Vandewalle et al.,^102^ the Oxymap system was used to study changes in retinal oxygen saturation in patients with glaucoma. A positive correlation was identified between glaucomatous damage (measured using visual field defects and retinal nerve fiber layer changes) and the oxygen saturation in retinal venules, whereas a negative correlation was found between glaucomatous damage and arteriovenous difference in oxygen saturation. As described above, two studies using hyperspectral imaging have also demonstrated increased oxygen saturation in retinal venules in patients with diabetic retinopathy.^58^^,^^61^ These findings may be due to reduced retinal oxygen consumption in glaucoma. Oximetry may also provide further insight into the changes observed after glaucoma surgery. For example, a study by Hardarson et al.^103^ found a small increase in the oxygen saturation of retinal arterioles of 19 patients following glaucoma filtration surgery. No significant changes were seen in venules, and the study ultimately concluded that glaucoma filtration surgery “had almost no effect” on retinal vessel oxygen saturation. As with other conditions, although retinal oximetry demonstrates promise, it has yet to demonstrate enough clinical utility to become a part of routine glaucoma practice.^104^

## Future Directions and Discussion

As described in this review, there have been many recent advances in the field of retinal oximetry. In addition to dual-wavelength imaging, which remains the most well-studied retinal oximetry technology, there are many emerging technologies that offer promise. From reviewing the literature, it is clear that oxygen saturation levels exhibit changes in a variety of ocular and systemic conditions. Continuing to characterize and understand these pathologic changes in retinal oxygen saturation will likely be important in understanding the disease pathophysiology.

Despite these promising findings, the numerous limitations of current techniques have prevented retinal oximetry from gaining widespread adoption as a standard clinical marker. Most importantly, although changes in retinal oxygen saturation have been demonstrated in a variety of clinical conditions, these findings have yet to demonstrate applications in which retinal oxygen saturation can reliably impact clinical decision-making. Two major limitations, described above, are the influence of fundus pigmentation and vessel size on retinal oximetry measurements. The ability to measure oxygen saturation in a diverse group of patients must be demonstrated prior to widespread adoption of this technology. The inability of current oximeters to scan beyond the inner retinal vasculature also remains a large limitation. Technologies capable of precisely measuring choroidal blood flow as well as all layers of the retina will certainly expand the applicability of these devices. In addition, the ability to measure the oxygen saturation of areas without vessels, such as the fovea, may allow one to obtain valuable information across the macula or around areas of geographic atrophy, microaneurysms, etc. However, given that the multi-wavelength technique relies on the absorption profile of hemoglobin, which is found within blood vessels, measuring oxygen saturation outside of blood vessels remains a challenge that will necessitate the use of additional techniques.

On the technical side, improvements are necessary to help overcome other limiting issues, including challenges in calibration, image processing algorithms, and the length of time that current devices require for image capture. Recent work from DePaoli et al.^105^ demonstrated that usage of a convolutional neural network for spectroscopic analysis can improve calculation performance when compared with existing algorithms. Improving image processing algorithms to improve the ease of use for untrained users will likely increase adoption. Furthermore, the large size of the majority of current products prevents measurement in children and bedridden patients. The Corimap handheld camera, which uses the dual-wavelength approach, aims to overcome this limitation, and future handheld products will be important to make this technology useful in this critical subset of patients.^106^ In addition, newer technologies that use several wavelengths, including those reviewed above, may be able to provide more accurate measurements compared to dual-wavelength products.

In conclusion, there has been considerable progress in the field of retinal oximetry over the past decade. Commercially available dual-wavelength systems have provided a wealth of data for healthy subjects, as well as a variety of disease states. At the same time, several promising technologies have emerged in an attempt to provide more accurate measurements. This research has shown that retinal oxygen saturation is affected in a variety of disease states. However, the various limitations of the current technologies, as well as a lack of clinical applicability, have prevented the widespread adoption of retinal oximetry in a clinical setting. Future work will require demonstrating a correlation of retinal oxygen saturation with disease progression or the ability to predict treatment response in order for retinal oximetry to progress toward becoming a standard clinical measurement.

## References

[1] Patel V, Rassam S, Newsom R, Wiek J, Kohner E Retinal blood flow in diabetic retinopathy. *BMJ*. 1992; 305(6855): 678–683. 1393111 10.1136/bmj.305.6855.678 PMC1882919

[2] Yoshida A, Feke GT, Morales-Stoppello J, Collas GD, Goger DG, McMeel JW. Retinal blood flow alterations during progression of diabetic retinopathy. *Arch Ophthalmol*. 1983; 101(2): 225–227. 6337586 10.1001/archopht.1983.01040010227008

[3] Friedman E, Krupsky S, Lane AM, et al. Ocular blood flow velocity in age-related macular degeneration. *Ophthalmology*. 1995; 102(4): 640–646. 7724181 10.1016/s0161-6420(95)30974-8

[4] Christoffersen NLB, Larsen M Pathophysiology and hemodynamics of branch retinal vein occlusion. *Ophthalmology*. 1999; 106(11): 2054–2062. 10571337 10.1016/S0161-6420(99)90483-9

[5] Flammer J, Orgül S, Costa VP, et al. The impact of ocular blood flow in glaucoma. *Prog Retin Eye Res*. 2002; 21(4): 359–393. 12150988 10.1016/s1350-9462(02)00008-3

[6] Chan ED, Chan MM, Chan MM Pulse oximetry: understanding its basic principles facilitates appreciation of its limitations. *Respir Med*. 2013; 107(6): 789–799. 23490227 10.1016/j.rmed.2013.02.004

[7] Beach J Pathway to retinal oximetry. *Transl Vis Sci Technol*. 2014; 3(5): 2. 10.1167/tvst.3.5.2 PMC4164112 25237591

[8] Hardarson SH, Harris A, Karlsson RA, et al. Automatic retinal oximetry. *Invest Ophthalmol Vis Sci*. 2006; 47(11): 5011–5016. 17065521 10.1167/iovs.06-0039

[9] Beach JM, Schwenzer KJ, Srinivas S, Kim D, Tiedeman JS Oximetry of retinal vessels by dual-wavelength imaging: calibration and influence of pigmentation. *J Appl Physiol*. 1999; 86(2): 748–758. 9931217 10.1152/jappl.1999.86.2.748

[10] Hickam JB, Sieker HO, Frayser R Studies of retinal circulation and A-V oxygen difference in man. *Trans Am Clin Climatol Assoc*. 1959; 71(657): 34–44. 14401681 PMC2248999

[11] Hickam JB, Frayser R Studies of the retinal circulation in man. Observations on vessel diameter, arteriovenous oxygen difference, and mean circulation time. *Circulation*. 1966; 33(2): 302–316. 25823104 10.1161/01.cir.33.2.302

[12] Delori FC Noninvasive technique for oximetry of blood in retinal vessels. *Appl Opt*. 1988; 27(6): 1113–1125. 20531526 10.1364/AO.27.001113

[13] Drewes JJ, Smith MH, Denninghoff KR, Hillman LW Instrument for the measurement of retinal vessel oxygen saturation. *Ophthalmic Technol IX*. 1999; 3591: 114.

[14] Geirsdottir A, Palsson O, Hardarson SH, Olafsdottir OB, Kristjansdottir JV, Stefánsson E Retinal vessel oxygen saturation in healthy individuals. *Invest Ophthalmol Vis Sci*. 2012; 53(9): 5433–5442. 22786895 10.1167/iovs.12-9912

[15] Hardarson SH, Stefánsson E Retinal oxygen saturation is altered in diabetic retinopathy. *Br J Ophthalmol*. 2012; 96(4): 560–563. 22080478 10.1136/bjophthalmol-2011-300640

[16] Olafsdottir OB, Hardarson SH, Gottfredsdottir MS, Harris A, Stefánsson E Retinal oximetry in primary open-angle glaucoma. *Invest Ophthalmol Vis Sci*. 2011; 52(9): 6409–6413. 21715353 10.1167/iovs.10-6985

[17] Olafsdottir OB, Vandewalle E, Abegão Pinto L, et al. Retinal oxygen metabolism in healthy subjects and glaucoma patients. *Br J Ophthalmol*. 2014; 98(3): 329–333. 24403567 10.1136/bjophthalmol-2013-303162

[18] Eysteinsson T, Hardarson SH, Bragason D, Stefánsson E Retinal vessel oxygen saturation and vessel diameter in retinitis pigmentosa. *Acta Ophthalmol*. 2014; 92(5): 449–453. 24767302 10.1111/aos.12359

[19] Zong Y, Lin L, Yi C, et al. Retinal vessel oxygen saturation and vessel diameter in retinitis pigmentosa at various ages. *Graefes Arch Clin Exp Ophthalmol*. 2016; 254(2): 243–252. 25952041 10.1007/s00417-015-3039-6

[20] Hardarson SH, Stefánsson E Oxygen saturation in central retinal vein occlusion. *Am J Ophthalmol*. 2010; 150(6): 871–875. 20875633 10.1016/j.ajo.2010.06.020

[21] Chen H, Lin H, Chen W, et al. Preoperative and postoperative measurements of retinal vessel oxygen saturation in patients with different grades of cataracts. *Acta Ophthalmol*. 2017; 95(6): e436–e442. 27864880 10.1111/aos.13332

[22] Geirsdottir A, Hardarson SH, Olafsdottir OB, Stefánsson E Retinal oxygen metabolism in exudative age-related macular degeneration. *Acta Ophthalmol*. 2014; 92(1): 27–33. 24447786 10.1111/aos.12294

[23] Long C, Wen X, Zhong L-X-Y, Zheng Y, Gao Q Oxygen saturation in closed-globe blunt ocular trauma. *Biomed Res Int*. 2016; 2016: 8232468. 27699174 10.1155/2016/8232468 PMC5028796

[24] Svrčinová T, Mareš J, Chrapek O, et al. Changes in oxygen saturation and the retinal nerve fibre layer in patients with optic neuritis - a pilot study. *Acta Ophthalmol*. 2018; 96(3): e309–e314. 29090843 10.1111/aos.13571

[25] Nitta E, Hirooka K, Shimazaki T, et al. Retinal oxygen saturation before and after glaucoma surgery. *Acta Ophthalmol*. 2017; 95(5): e350–e353. 27775227 10.1111/aos.13274

[26] Li Z, Zhang J, Lin T, Peng W, Lu L, Hu J Macular vascular circulation and retinal oxygen saturation changes for idiopathic macular epiretinal membrane after vitrectomy. *Acta Ophthalmol*. 2019; 97(3): 296–302. 30843354 10.1111/aos.14066

[27] Zheng Q, Zong Y, Li L, et al. Retinal vessel oxygen saturation and vessel diameter in high myopia. *Ophthalmic Physiol Opt*. 2015; 35(5): 562–569. 26303449 10.1111/opo.12223

[28] Yang X, Huang D, Ai S, Liang X, Zhao J, Fang L Retinal vessel oxygen saturation and vessel diameter in inactive graves ophthalmopathy. *Ophthalmic Plast Reconstr Surg*. 2017; 33(6): 459–465. 27893583 10.1097/IOP.0000000000000826

[29] Hafner J, Ginner L, Karst S, et al. Regional patterns of retinal oxygen saturation and microvascular hemodynamic parameters preceding retinopathy in patients with type II diabetes. *Invest Ophthalmol Vis Sci*. 2017; 58(12): 5541–5547. 29075765 10.1167/iovs.17-22523

[30] Eliasdottir TS, Bragason D, Hardarson SH, et al. Retinal oximetry measures systemic hypoxia in central nervous system vessels in chronic obstructive pulmonary disease. *PLoS One*. 2017; 12(3): e0174026. 28328974 10.1371/journal.pone.0174026 PMC5362093

[31] Willerslev A, Hansen MM, Klefter ON, et al. Non-invasive imaging of retinal blood flow in myeloproliferative neoplasms. *Acta Ophthalmol*. 2017; 95(2): 146–152. 27682603 10.1111/aos.13249

[32] Einarsdottir AB, Hardarson SH, Kristjansdottir JV, Bragason DT, Snaedal J, Stefánsson E Retinal oximetry imaging in Alzheimer's disease. *J Alzheimers Dis*. 2016; 49(1): 79–83. 26444785 10.3233/JAD-150457

[33] Einarsdottir AB, Olafsdottir OB, Hjaltason H, Hardarson SH Retinal oximetry is affected in multiple sclerosis. *Acta Ophthalmol*. 2018; 96(5): 528–530. 29338134 10.1111/aos.13682

[34] Palsson O, Geirsdottir A, Hardarson SH, Olafsdottir OB, Kristjansdottir JV, Stefánsson E Retinal oximetry images must be standardized: a methodological analysis. *Invest Ophthalmol Vis Sci*. 2012; 53(4): 1729–1733. 22395877 10.1167/iovs.11-8621

[35] Kristjansdottir JV, Hardarson SH, Harvey AR, Olafsdottir OB, Eliasdottir TS, Stefánsson E Choroidal oximetry with a noninvasive spectrophotometric oximeter. *Invest Ophthalmol Vis Sci*. 2013; 54(5): 3234–3239. 23572109 10.1167/iovs.12-10507

[36] Nakano Y, Shimazaki T, Kobayashi N, et al. Retinal oximetry in a healthy Japanese population. *PLoS One*. 2016; 11(7): e0159650. 27434373 10.1371/journal.pone.0159650 PMC4951009

[37] Liu X, Wang S, Liu Y, et al. Retinal oxygen saturation in Chinese adolescents. *Acta Ophthalmol*. 2017; 95(1): e54–e61. 27807947 10.1111/aos.13167

[38] Mohan A, Dabir S, Yadav NK, Kummelil M, Kumar RS, Shetty R Normative database of retinal oximetry in Asian Indian eyes. *PLoS One*. 2015; 10(4): e0126179. 25923699 10.1371/journal.pone.0126179 PMC4414619

[39] Garhofer G, Bek T, Boehm AG, et al. Use of the retinal vessel analyzer in ocular blood flow research. *Acta Ophthalmol*. 2010; 88(7): 717–722. 19681764 10.1111/j.1755-3768.2009.01587.x

[40] Türksever C, Orgül S, Todorova MG Reproducibility of retinal oximetry measurements in healthy and diseased retinas. *Acta Ophthalmol*. 2015; 93(6): e439–45. 25430037 10.1111/aos.12598

[41] Narasimha-Iyer H, Mahadevan V, Beach JM, Roysam B Improved detection of the central reflex in retinal vessels using a generalized dual-Gaussian model and robust hypothesis testing. *IEEE Trans Inf Technol Biomed*. 2008; 12(3): 406–410. 18693508 10.1109/titb.2007.897782

[42] Blair NP, Wanek J, Felder AE, et al. Retinal oximetry and vessel diameter measurements with a commercially available scanning laser ophthalmoscope in diabetic retinopathy. *Invest Ophthalmol Vis Sci*. 2017; 58(12): 5556–5563. 29079858 10.1167/iovs.17-21934 PMC5656420

[43] Hammer M, Vilser W, Riemer T, Schweitzer D Retinal vessel oximetry-calibration, compensation for vessel diameter and fundus pigmentation, and reproducibility. *J Biomed Opt*. 2008; 13(5): 054015. 19021395 10.1117/1.2976032

[44] Liu W, Jiao S, Zhang HF Accuracy of retinal oximetry: a Monte Carlo investigation. *J Biomed Opt*. 2013; 18(6): 066003. 23733019 10.1117/1.JBO.18.6.066003 PMC3669519

[45] Rodriguez DA, Pfefer TJ, Wang Q, Lopez PF, Ramella-Roman JC A Monte Carlo analysis of error associated with two-wavelength algorithms for retinal oximetry. *Invest Ophthalmol Vis Sci*. 2016; 57(14): 6474–6481. 27893889 10.1167/iovs.16-20138

[46] Waizel M, Türksever C, Todorova MG Normative values of retinal vessel oximetry in healthy children against adults. *Acta Ophthalmol*. 2018; 96(7): e828–e834. 30187646 10.1111/aos.13726

[47] Mohan A, Dabir S, Kurian M, Shetty R, Chidambara L, Kumar RS Perivascular and quadrant nerve fiber layer thickness and its relationship with oxygen saturation. *Curr Eye Res*. 2016; 41(9): 1223–1228. 26765040 10.3109/02713683.2015.1104361

[48] Patel SR, Hudson C, Flanagan JG, Heitmar R The effect of simulated cataract light scatter on retinal vessel oximetry. *Exp Eye Res*. 2013; 116: 185–189. 24056207 10.1016/j.exer.2013.09.004

[49] Jeppesen SK, Bek T The retinal oxygen saturation measured by dual wavelength oximetry in larger retinal vessels is influenced by the linear velocity of the blood. *Curr Eye Res*. 2019; 44(1): 46–52. 30230380 10.1080/02713683.2018.1524015

[50] Vasefi F, MacKinnon N, Farkas DL. Hyperspectral and multispectral imaging in dermatology. In: Hamblin M, Avci P, Gupta G, eds. *Imaging in Dermatology*. Amsterdam: Elsevier; 2016: 187–201.

[51] Lu G, Fei B Medical hyperspectral imaging: a review. *J Biomed Opt*. 2014; 19(1): 10901. 24441941 10.1117/1.JBO.19.1.010901 PMC3895860

[52] Cohen D, Arnoldussen M, Bearman G, Grundfest WS Use of spectral imaging for the diagnosis of retinal disease. In: *Conference Proceedings - Lasers and Electro-Optics Society Annual Meeting-LEOS*. Piscataway, NJ: Institute of Electrical and Electronics Engineers; 1999: 220–221.

[53] Kaluzny J, Li H, Liu W, et al. Bayer filter snapshot hyperspectral fundus camera for human retinal imaging. *Curr Eye Res*. 2017; 42(4): 629–635. 27767345 10.1080/02713683.2016.1221976 PMC5389919

[54] Patel SR, Flanagan JG, Shahidi AM, Sylvestre J-P, Hudson C A prototype hyperspectral system with a tunable laser source for retinal vessel imaging. *Invest Ophthalmol Vis Sci*. 2013; 54(8): 5163–5168. 23821191 10.1167/iovs.13-12124

[55] Desjardins M, Sylvestre J-P, Jafari R, et al. Preliminary investigation of multispectral retinal tissue oximetry mapping using a hyperspectral retinal camera. *Exp Eye Res*. 2016; 146: 330–340. 27060375 10.1016/j.exer.2016.04.001

[56] Mordant DJ, Al-Abboud I, Muyo G, et al. Spectral imaging of the retina. *Eye (Lond)*. 2011; 25(3): 309–320. 21390065 10.1038/eye.2010.222 PMC3178323

[57] Dwight JG, Weng CY, Coffee RE, Pawlowski ME, Tkaczyk TS Hyperspectral image mapping spectrometry for retinal oximetry measurements in four diseased eyes. *Int Ophthalmol Clin*. 2016; 56(4): 25–38. 27575756 10.1097/IIO.0000000000000139 PMC5103700

[58] Mordant DJ, Al-Abboud I, Muyo G, Gorman A, Harvey AR, McNaught AI Oxygen saturation measurements of the retinal vasculature in treated asymmetrical primary open-angle glaucoma using hyperspectral imaging. *Eye (Lond)*. 2014; 28(10): 1190–1200. 25060843 10.1038/eye.2014.169 PMC4194338

[59] Rose K, Krema H, Durairaj P, et al. Retinal perfusion changes in radiation retinopathy. *Acta Ophthalmol*. 2018; 96(6): e727–e731. 29998553 10.1111/aos.13797

[60] Tayyari F, Khuu L-A, Flanagan JG, Singer S, Brent MH, Hudson C Retinal blood flow and retinal blood oxygen saturation in mild to moderate diabetic retinopathy. *Invest Ophthalmol Vis Sci*. 2015; 56(11): 6796–6800. 26567792 10.1167/iovs.15-17481

[61] Shahidi AM, Hudson C, Tayyari F, Flanagan JG Retinal oxygen saturation in patients with primary open-angle glaucoma using a non-flash hypespectral camera. *Curr Eye Res*. 2017; 42(4): 557–561. 27612016 10.1080/02713683.2016.1217544

[62] Laufer J, Elwell C, Delpy D, Beard P In vitro measurements of absolute blood oxygen saturation using pulsed near-infrared photoacoustic spectroscopy: accuracy and resolution. *Phys Med Biol*. 2005; 50(18): 4409–4428. 16148401 10.1088/0031-9155/50/18/011

[63] Zhang HF, Maslov K, Stoica G, Wang LV Functional photoacoustic microscopy for high-resolution and noninvasive in vivo imaging. *Nat Biotechnol*. 2006; 24(7): 848–851. 16823374 10.1038/nbt1220

[64] Liu W, Zhang HF Photoacoustic imaging of the eye: a mini review. *Photoacoustics*. 2016; 4(3): 112–123. 27761410 10.1016/j.pacs.2016.05.001 PMC5063360

[65] de la Zerda A, Paulus YM, Teed R, et al. Photoacoustic ocular imaging. *Opt Lett*. 2010; 35(3): 270. 20125691 10.1364/OL.35.000270 PMC2886805

[66] Liu T, Li H, Song W, Jiao S, Zhang HF Fundus camera guided photoacoustic ophthalmoscopy. *Curr Eye Res*. 2013; 38(12): 1229–1234. 24131226 10.3109/02713683.2013.815219 PMC3986591

[67] Liu W, Schultz KM, Zhang K, et al. In vivo corneal neovascularization imaging by optical-resolution photoacoustic microscopy. *Photoacoustics*. 2014; 2(2): 81–86. 25013754 10.1016/j.pacs.2014.04.003 PMC4083229

[68] Song W, Wei Q, Liu W, et al. A combined method to quantify the retinal metabolic rate of oxygen using photoacoustic ophthalmoscopy and optical coherence tomography. *Sci Rep*. 2014; 4: 6525. 25283870 10.1038/srep06525 PMC4185377

[69] Jiao S, Jiang M, Hu J, et al. Photoacoustic ophthalmoscopy for in vivo retinal imaging. *Opt Express*. 2010; 18(4): 3967–3972. 20389409 10.1364/OE.18.003967 PMC2864517

[70] Hennen SN, Xing W, Shui Y-B, et al. Photoacoustic tomography imaging and estimation of oxygen saturation of hemoglobin in ocular tissue of rabbits. *Exp Eye Res*. 2015; 138: 153–158. 26048477 10.1016/j.exer.2015.05.022 PMC5821107

[71] Hariri A, Wang J, Kim Y, Jhunjhunwala A, Chao DL, Jokerst JV In vivo photoacoustic imaging of chorioretinal oxygen gradients. *J Biomed Opt*. 2018; 23(3): 1–8. 10.1117/1.JBO.23.3.036005 PMC5844348 29524321

[72] Wang LV, Hu S Photoacoustic tomography: in vivo imaging from organelles to organs. *Science*. 2012; 335(6075): 1458–1462. 22442475 10.1126/science.1216210 PMC3322413

[73] Huang D, Swanson EA, Lin CP, et al. Optical coherence tomography. *Science*. 1991; 254(5035): 1178–1181. 1957169 10.1126/science.1957169 PMC4638169

[74] Považay B et al. Visible light optical coherence tomography. *Proc SPIE*. 2002; 4619, 90–94.

[75] Chong SP, Bernucci M, Radhakrishnan H, Srinivasan VJ Structural and functional human retinal imaging with a fiber-based visible light OCT ophthalmoscope. *Biomed Opt Express*. 2017; 8(1): 323–337. 28101421 10.1364/BOE.8.000323 PMC5231302

[76] Yi J, Wei Q, Liu W, Backman V, Zhang HF Visible-light optical coherence tomography for retinal oximetry. *Opt Lett*. 2013; 38(11): 1796–1798. 23722747 10.1364/OL.38.001796 PMC3986589

[77] Chen S, Yi J, Zhang HF Measuring oxygen saturation in retinal and choroidal circulations in rats using visible light optical coherence tomography angiography. *Biomed Opt Express*. 2015; 6(8): 2840–2853. 26309748 10.1364/BOE.6.002840 PMC4541512

[78] Yi J, Chen S, Shu X, Fawzi AA, Zhang HF Human retinal imaging using visible-light optical coherence tomography guided by scanning laser ophthalmoscopy. *Biomed Opt Express*. 2015; 6(10): 3701–3713. 26504622 10.1364/BOE.6.003701 PMC4605031

[79] Chen S, Shu X, Nesper PL, Liu W, Fawzi AA, Zhang HF Retinal oximetry in humans using visible-light optical coherence tomography [invited]. *Biomed Opt Express*. 2017; 8(3): 1415–1429. 28663838 10.1364/BOE.8.001415 PMC5480553

[80] Pi S, Hormel TT, Wei X, et al. Retinal capillary oximetry with visible light optical coherence tomography. *Proc Natl Acad Sci USA*. 2020; 117(21). 10.1073/pnas.1918546117 PMC7260968 32398376

[81] Song W, Shao W, Yi W, et al. Visible light optical coherence tomographyangiography (vis-OCTA) facilitates localmicrovascular oximetry in the human retina. *Biomed Opt Express*. 2020; 11(7): 4037–4051. 33014584 10.1364/BOE.395843 PMC7510897

[82] Shah RS, Soetikno BT, Yi J, et al. Visible-light optical coherence tomography angiography for monitoring laser-induced choroidal neovascularization in mice. *Invest Ophthalmol Vis Sci*. 2016; 57(9): OCT86–OCT95. 27409510 10.1167/iovs.15-18891 PMC4968775

[83] Shu X, Beckmann L, Wang Y, et al. Designing visible-light optical coherence tomography towards clinics. *Quant Imaging Med Surg*. 2019; 9(5): 769–781. 31281773 10.21037/qims.2019.05.01 PMC6571199

[84] Brown MM, Brown GC, Lieske HB, Tran I, Turpcu A, Colman S Societal costs associated with neovascular age-related macular degeneration in the United States. *Retina*. 2016; 36(2): 285–298. 26428606 10.1097/IAE.0000000000000717

[85] Jakobsen DB, Torp TL, Stefansson E, Peto T, Grauslund J Retinal metabolic and structural alterations in response to aflibercept treatment in neovascular age-related macular degeneration. *Acta Ophthalmol*. 2019; 97(5): 525–531. 30549221 10.1111/aos.13996

[86] Tarr JM, Kaul K, Chopra M, Kohner EM, Chibber R Pathophysiology of diabetic retinopathy. *ISRN Ophthalmol*. 2013; 2013: 343560. 24563789 10.1155/2013/343560 PMC3914226

[87] Whitehead M, Wickremasinghe S, Osborne A, Van Wijngaarden P, Martin KR Diabetic retinopathy: a complex pathophysiology requiring novel therapeutic strategies. *Expert Opin Biol Ther*. 2018; 18(12): 1257–1270. 30408422 10.1080/14712598.2018.1545836 PMC6299358

[88] Hammer M, Vilser W, Riemer T, et al. Diabetic patients with retinopathy show increased retinal venous oxygen saturation. *Graefes Arch Clin Exp Ophthalmol*. 2009; 247(8): 1025–1030. 19404666 10.1007/s00417-009-1078-6

[89] Bek T, Stefánsson E, Hardarson SH Retinal oxygen saturation is an independent risk factor for the severity of diabetic retinopathy. *Br J Ophthalmol*. 2019; 103(8): 1167–1172. 30337331 10.1136/bjophthalmol-2018-312764

[90] Kashani AH, Lopez Jaime GR, Saati S, Martin G, Varma R, Humayun MS Noninvasive assessment of retinal vascular oxygen content among normal and diabetic human subjects: a study using hyperspectral computed tomographic imaging spectroscopy. *Retina*. 2014; 34(9): 1854–1860. 24732694 10.1097/IAE.0000000000000146 PMC4145024

[91] Bek T, Jørgensen CM The systemic blood pressure and oxygen saturation in retinal arterioles predict the effect of intravitreal anti-VEGF treatment on diabetic maculopathy. *Invest Ophthalmol Vis Sci*. 2016; 57(13): 5429–5434. 27756077 10.1167/iovs.16-20305

[92] Jørgensen CM, Hardarson SH, Bek T The oxygen saturation in retinal vessels from diabetic patients depends on the severity and type of vision-threatening retinopathy. *Acta Ophthalmol*. 2014; 92(1): 34–39. 24330421 10.1111/aos.12283

[93] Jørgensen C, Bek T Increasing oxygen saturation in larger retinal vessels after photocoagulation for diabetic retinopathy. *Invest Ophthalmol Vis Sci*. 2014; 55(8): 5365–5369. 25097242 10.1167/iovs.14-14811

[94] Laouri M, Chen E, Looman M, Gallagher M The burden of disease of retinal vein occlusion: review of the literature. *Eye (Lond)*. 2011; 25(8): 981–988. 21546916 10.1038/eye.2011.92 PMC3178209

[95] Yang J-Y, You B, Wang Q, Chan SY, Jonas JB, Wei W-B Retinal vessel oxygen saturation in healthy subjects and early branch retinal vein occlusion. *Int J Ophthalmol*. 2017; 10(2): 267–270. 28251087 10.18240/ijo.2017.02.14 PMC5313551

[96] Hardarson SH, Stefánsson E Oxygen saturation in branch retinal vein occlusion. *Acta Ophthalmol*. 2012; 90(5): 466–470. 21518303 10.1111/j.1755-3768.2011.02109.x

[97] Šínová I, Řehák J, Nekolová J, et al. Correlation between ischemic index of retinal vein occlusion and oxygen saturation in retinal vessels. *Am J Ophthalmol*. 2018; 188: 74–80. 29366614 10.1016/j.ajo.2018.01.015

[98] Nicholson L, Vazquez-Alfageme C, Hykin PG, Bainbridge JW, Sivaprasad S The relationship between retinal vessel oxygenation and spatial distribution of retinal nonperfusion in retinal vascular diseases. *Invest Ophthalmol Vis Sci*. 2019; 60(6): 2083–2087. 31091315 10.1167/iovs.18-24917

[99] Moore D, Harris A, Wudunn D, Kheradiya N, Siesky B Dysfunctional regulation of ocular blood flow: a risk factor for glaucoma? *Clin Ophthalmol*. 2008; 2(4): 849–861. 19668439 10.2147/opth.s2774 PMC2699797

[100] Flammer J, Mozaffarieh M What is the present pathogenetic concept of glaucomatous optic neuropathy? *Surv Ophthalmol*. 2007; 52(suppl 2): S162–S173. 17998042 10.1016/j.survophthal.2007.08.012

[101] Bonomi L, Marchini G, Marraffa M, Bernardi P, Morbio R, Varotto A Vascular risk factors for primary open angle glaucoma: the Egna-Neumarkt Study. *Ophthalmology*. 2000; 107(7): 1287–1293. 10889099 10.1016/s0161-6420(00)00138-x

[102] Vandewalle E, Abegão Pinto L, Olafsdottir OB, et al. Oximetry in glaucoma: correlation of metabolic change with structural and functional damage. *Acta Ophthalmol*. 2014; 92(2): 105–110. 23323611 10.1111/aos.12011

[103] Hardarson SH, Gottfredsdottir MS, Halldorsson GH, et al. Glaucoma filtration surgery and retinal oxygen saturation. *Invest Ophthalmol Vis Sci*. 2009; 50(11): 5247–5250. 19494205 10.1167/iovs.08-3117

[104] Yap ZL, Verma S, Lee YF, Ong C, Mohla A, Perera SA Glaucoma related retinal oximetry: a technology update. *Clin Ophthalmol*. 2018; 12: 79–84. 29379268 10.2147/OPTH.S128459 PMC5757969

[105] DePaoli DT, Tossou P, Parent M, Sauvageau D, Côté DC Convolutional neural networks for spectroscopic analysis in retinal oximetry. *Sci Rep*. 2019; 9(1): 1–13. 31388136 10.1038/s41598-019-47621-7 PMC6684811

[106] Vehmeijer W, Hardarson SH, Jonkman K, et al. Handheld retinal oximetry in healthy young adults. *Transl Vis Sci Technol*. 2018; 7(4): 19. 10.1167/tvst.7.4.19 PMC6114026 30174997

[107] Hickam JB, Frayser R, Ross JC A study of retinal venous blood oxygen saturation in human subjects by photographic means. *Circulation*. 1963; 27: 375–385. 13961118 10.1161/01.cir.27.3.375

[108] Schweitzer D, Hammer M, Kraft J, Thamm E, Königsdörffer E, Strobel J In vivo measurement of the oxygen saturation of retinal vessels in healthy volunteers. *IEEE Trans Biomed Eng*. 1999; 46(12): 1454–1465. 10612903 10.1109/10.804573

[109] Kim SK, Kim DM, Suh MH, Kim M, Kim HC Retinal oximetry based on nonsimultaneous image acquisition using a conventional fundus camera. *IEEE Trans Med Imaging*. 2011; 30(8): 1577–1580. 21478073 10.1109/TMI.2011.2140329

[110] Heitmar R, Safeen S Regional differences in oxygen saturation in retinal arterioles and venules. *Graefes Arch Clin Exp Ophthalmol*. 2012; 250(10): 1429–1434. 22395204 10.1007/s00417-012-1980-1

[111] Man REK, Kawasaki R, Wu Z, et al. Reliability and reproducibility of retinal oxygen saturation measurements using a predefined peri-papillary annulus. *Acta Ophthalmol*. 2013; 91(8): e590–e594. 23802705 10.1111/aos.12173

[112] Shahidi AM, Patel SR, Flanagan JG, Hudson C Regional variation in human retinal vessel oxygen saturation. *Exp Eye Res*. 2013; 113: 143–147. 23791637 10.1016/j.exer.2013.06.001

[113] Jani PD, Mwanza J-C, Billow KB, Waters AM, Moyer S, Garg S Normative values and predictors of retinal oxygen saturation. *Retina*. 2014; 34(2): 394–401. 23842102 10.1097/IAE.0b013e3182979e7b

[114] Man REK, Sasongko MB, Kawasaki R, et al. Associations of retinal oximetry in healthy young adults. *Invest Ophthalmol Vis Sci*. 2014; 55(3): 1763–1769. 24526435 10.1167/iovs.13-13320

[115] Palkovits S, Lasta M, Told R, et al. Retinal oxygen metabolism during normoxia and hyperoxia in healthy subjects. *Invest Ophthalmol Vis Sci*. 2014; 55(8): 4707–4713. 25015353 10.1167/iovs.14-14593

[116] Yip W, Siantar R, Perera SA, et al. Reliability and determinants of retinal vessel oximetry measurements in healthy eyes. *Invest Ophthalmol Vis Sci*. 2014; 55(11): 7104–7110. 25301879 10.1167/iovs.13-13854

[117] Yang W, Fu Y, Dong Y, et al. Retinal vessel oxygen saturation in a healthy young Chinese population. *Acta Ophthalmol*. 2016; 94(4): 373–379. 26742652 10.1111/aos.12943

[118] Told R, Boltz A, Schmetterer L, et al. Method comparison of two non-invasive dual-wavelength spectrophotometric retinal oximeters in healthy young subjects during normoxia. *Acta Ophthalmol*. 2018; 96(5): e614–e618. 29488329 10.1111/aos.13719

[119] Choi W, Park E-Y, Jeon S, Kim C Clinical photoacoustic imaging platforms. *Biomed Eng Lett*. 2018; 8(2): 139–155. 30603199 10.1007/s13534-018-0062-7 PMC6208525

[120] Zhang T, Kho AM, Srinivasan VJ Improving visible light OCT of the human retina with rapid spectral shaping and axial tracking. *Biomed Opt Express*. 2019; 10(6): 2918–2931. 31259062 10.1364/BOE.10.002918 PMC6583340

[121] Eliasdottir TS Retinal oximetry and systemic arterial oxygen levels. *Acta Ophthalmol*. 2018; 96(suppl A113): 1–44. 10.1111/aos.13932 30460761

[122] Zijlstra WG, Buursma A, van Assendelft OW *Visible and Near Infrared Absorption Spectra of Human and Animal Haemoglobin : Determination and Application*. Boca Raton, FL : CRC Press; 2000.

[123] Yao J, Wang LV Photoacoustic microscopy. *Laser Photon Rev*. 2013; 7(5): 1–7. 10.1002/lpor.201200060 PMC3887369 24416085

[124] Pi S, Camino A, Cepurna W, et al. Automated spectroscopic retinal oximetry with visible-light optical coherence tomography. *Biomed Opt Express*. 2018; 9(5): 2056–2067. 29760969 10.1364/BOE.9.002056 PMC5946770

